# Hypermethylation in the promoter of the *MTHFR* gene is associated with diabetic complications and biochemical indicators

**DOI:** 10.1186/s13098-017-0284-3

**Published:** 2017-10-18

**Authors:** Mayara Karla dos Santos Nunes, Alexandre Sérgio Silva, Isabella Wanderley de Queiroga Evangelista, João Modesto Filho, Cecília Neta Alves Pegado Gomes, Rayner Anderson Ferreira do Nascimento, Rafaella Cristhine Pordeus Luna, Maria José de Carvalho Costa, Naila Francis Paulo de Oliveira, Darlene Camati Persuhn

**Affiliations:** 1 Post-Graduation Program in Cellular and Molecular Biology, Federal University of Paraiba, Joao Pessoa, Brazil; 2 Physical Education Department, Federal University of Paraiba, Joao Pessoa, Brazil; 3 Ophthalmology Reference Center, Lauro Wanderley University Hospital, Federal University of Paraiba, Joao Pessoa, Brazil; 4 Department of Internal Medicine, Federal University of Paraiba, Joao Pessoa, Brazil; 5 Nephrology Clinic, Lauro Wanderley University Hospital, Federal University of Paraiba, Joao Pessoa, Brazil; 6 Faculty Mauricio of Nassau, Joao Pessoa, Brazil; 7 Post-Graduate Program in Nutrition Science, Federal University of Paraiba, Joao Pessoa, Brazil; 8 Nutrition Science Department and Post-Graduate Program in Nutrition Science, Federal University of Paraiba, Joao Pessoa, Brazil; 9 Department of Molecular Biology, Federal University of Paraiba, Joao Pessoa, Brazil; 10 Department of Molecular Biology and Post-Graduation Program in Nutrition Science, Federal University of Paraiba, CEP 58051-900 Joao Pessoa, Brazil

## Abstract

**Background:**

DNA methylation is an epigenetic mechanism for regulating the transcription of many genes and has been linked to the development of various diseases. A promising gene to investigate is methylenetetrahydrofolate reductase (MTHFR), since the enzyme methylenetetrahydrofolate reductase (MTHFR) promotes methyl radical synthesis in the homocysteine cycle and can provide methyl groups for DNA methylation. In addition, several studies have correlated gene polymorphisms of this enzyme with a greater risk of diabetes, but little is known regarding the relationship between epigenetic changes in this gene and diabetes and its complications. The aim of this study was to investigate the relationship between methylation profile in the MTHFR gene promoter and biochemical, inflammatory and oxidative stress markers in individuals with type 2 diabetes (T2DM) who have been diagnosed for 5–10 years with or without diabetic retinopathy (DR) and nephropathy (DN).

**Methods:**

Specific PCR for methylation (MSP) was used to analyze MTHFR methylation profile in leucocytes DNA. Biochemical markers (glycemia, glycated hemoglobin, total cholesterol, LDL, HDL, triglycerides, serum creatinine), inflammatory markers (C-reactive protein and alpha-1 acid glycoprotein) and oxidative stress (total antioxidant and malonaldehyde) were determined in peripheric blood samples and microalbuminuria in 24 h urine samples. The X^2^ and Mann–Whitney statistical tests were performed and p < 0.05 were considered significant.

**Results:**

The hypermethylated profile was most frequently observed in individuals with retinopathy (p < 0.01) and was associated with higher total cholesterol and LDL levels (p = 0.0046, 0.0267, respectively). Individuals with DN and hypermethylated profiles had higher levels of alpha-1 acid glycoprotein (p = 0.0080) and total antioxidant capacity (p = 0.0169) compared to subjects without complications.

**Conclusions:**

Hypermethylation in the promoter of the MTHFR gene is associated with the occurrence of DR and with biochemical, inflammatory and oxidative stress parameters in the context of chronic complications

## Background

DNA methylation is an epigenetic mechanism that regulates the transcription of many genes by preventing transcription factor binding or promoting the binding of methyl binding proteins (MBP) causing inhibition or decreased gene expression [1]. The methyl group bound to a cytosine that precedes a guanine (CpG dinucleotide) can be donated by the homocysteine (Hcy) cycle reactions, generated from methionine metabolism, and can be methylated by the enzyme 5,10-methylenetetrahydrofolate reductase (*MTHFR*) to form 5-methyltetrahydrofolate. In this way, methionine is converted into a universal methyl radical donor, the S-adenosylmethionine (SAMe) [2, 3].

As DNA methylation involves the transfer of a methyl group to a cytosine base, *MTHFR* is important in this mechanism; several studies have correlated gene polymorphisms in that enzyme with risk for the development of cancer, vascular diseases and diabetes [4, 5]. Changes in the methylation profile may lead to changes in enzyme expression and may also cause dysregulation of homocysteine metabolism [4].

Diabetes mellitus (DM) is currently a major public health problem worldwide due to its complications because it is one of the major causes of death and hospitalization and because it demands large expenditures from governments [6]. The most frequent form is type 2 diabetes mellitus (T2DM) and several studies suggest a multifactorial cause [7]. The clinical progression of poorly controlled diabetes results in hyperglycemia, ketoacidosis and nonketotic hyperosmolar coma [8]. Chronic complications that are characterized by micro- and macrovascular changes develop as a result of prolonged hyperglycemia. These complications consist of cardiovascular problems, neuropathy, retinopathy (DR) and nephropathy (DN) [7].

DN is a serious microvascular complication of DM and has become the major cause of end-stage renal failure. DR is the leading cause of blindness in adults [7]. Several studies point to the relationship between Hcy levels (hyperhomocysteinemia) and insulin resistance, which is the main cause of diabetic complications, such as DR and DN, in T2DM and cardiovascular diseases, especially when the polymorphism *MTHFR* C677T is present [8, 9]. An elevation in plasma levels of Hcy results in an increase in oxidative stress inducing an increase in pro-inflammatory cytokines, endothelial dysfunction, DNA damage and hypomethylation [10, 11].

There is growing evidence that epigenetic dysregulation is involved in diabetes and its associated DN and DR [12–17]. In addition, epigenetic alterations and the C677T genotype were associated with risk for chronic renal failure and dialysis in patients with renal insufficiency due to low enzyme activity and global hypomethylation of DNA that alters the expression of different systemic alleles [18]. While the relationship of *MTHFR* gene polymorphisms with T2DM is well-documented in the literature, little is known regarding the methylation profile of the *MTHFR* gene in this disease [5, 19]. This gene’s promoter region has two CpG islands with binding sites for various transcription factors, suggesting that DNA methylation plays an important role in the regulation of is transcription [20].

Based on these facts, the objective of this study was to investigate the relationship between the methylation profile of the *MTHFR* gene promoter and the occurrence of microvascular complications (DR and DN) in patients with diabetes for 5 and 10 years, which is the period when chronic complications usually begin. In addition, this study investigates the relationship of the biochemical, inflammatory and oxidative stress parameters to the methylation profile.

## Methods

### Subject recruitment and study logistics

The study consisted of 105 individuals found in the Reference Services for the diabetic patient of the Lauro Wanderley University Hospital of the Federal University of Paraiba (HULW/UFPB) from December 2013 to November 2016. Patients with T2DM of both sexes aged more than 40 years old with at least 5 and at most 10 years of disease evolution with or without diabetic complications were included. The subjects were divided into: CONTROL (n = 60)—diabetic patients without complications; DR (n = 16)—diabetic patients with retinopathy and DN (n = 29)—diabetic nephropathy patients.

### Ethical aspects

The study was approved by the Ethics Committee for Human Research of the Lauro Wanderley University Hospital from the Federal University of Paraíba (Opinion: 424.423/2013). In addition, all procedures followed were in accordance with the institution’s ethical standards, conducted in compliance with Resolution 466/2012 of the National Health Council and the International Declaration of Helsinki.

### Collection of biological samples

Blood was collected from all volunteers after a 12 h fast, and urine was collected at 24 h. A form containing clinical variables of the participants was completed.

For biochemical analysis, the blood was collected by means of venous puncture 3 different sterile tubes: tube 1 (with anticoagulant K_3_ EDTA), tube 2 (with anticoagulant sodium fluoride) and tube 3 (with clot activator). All samples from tubes 2 and 3 were immediately centrifuged to obtain plasma and serum, respectively and subjected to analysis within 2 h after collection.

For DNA extraction, blood collection was performed to obtain leukocytes by venous puncture in sterile tubes containing 7.2 mg of K_3_ EDTA.

### Biochemical analyses

To determine the biochemical properties of each sample: glycemia, total cholesterol, HDL cholesterol, triglycerides was measured using the enzymatic method, glycated hemoglobin (HbA_1_C), C-reactive protein (CRP) and alpha-1 acid glycoprotein (AGP) were measured using the immunoturbidimetry technique, serum creatinine was measured using a colorimetric method, and urinary albumin was analyzed with the turbidimetric technique. All tests were performed in an automated analyzer (LabMax 240, Labtest, Lagoa Santa, MG, Brazil) using standardized kit following the instructions provided by the manufacturer (Labtest, Lagoa Santa, MG, Brazil). The LDL concentration was determined by the Friedewald formula, where [LDL] = [total cholesterol] − [HDL − [triglycerides ÷ 5]. The markers of oxidative stress, malondialdehyde (MDA) and total antioxidant capacity (TAC), were determined by techniques already established in the literature [21, 22].

### Analysis of the methylation profile

#### DNA extraction

The blood samples were diluted in a lysis solution containing 10 mM Tris–HCl pH 8, 5 mM EDTA, 0.3 M sucrose, 1% Triton-X-100. This step was followed by centrifugation at 3200 rpm to discard the supernatant. This process was repeated 3 times in order to obtain a leukocyte precipitate free from hemoglobin remnants. The precipitate was later resuspended in lysis solution containing 10 mM Tris–HCl pH 8, sodium dodecyl sulfate (SDS) 0.5%, 5 mM EDTA and 0.2 μg proteinase K (Invitrogen, Carlsbad, CA, USA) and incubated at 55 °C in a water bath. After 7 h of incubation, 500 μl of an aqueous solution of 1 mM EDTA and 7.5 M ammonium acetate was added. The mixture was centrifuged for 10 min at 14,000*g* at 4 °C and 700 μl of the supernatant was transferred to a new tube where DNA precipitation was performed with 540 μl of isopropanol. Next, the DNA precipitate was washed with 70% ethanol, centrifuged (12,000*g* for 5 min), dried and resuspended in Tris–EDTA pH 8.0 buffer [23].

#### Specific PCR for methylation (MSP)

The previously extracted leukocyte DNA was converted (500 ng) by sodium bisulfite, which transforms unmethylated cytosine into uracil without changing methylated cytosine [24] from the EZ DNA Methylation™ Kit (ZymoResearch) according to the manufacturer’s instructions.

For each methylation-specific PCR reaction, 100 ng of DNA was bisulfite-transformed, 0.7 μl (7 μM) of each specific primer for methylated targets (sense: 5′-tagatttaggtacgtgaagtagggtagac-3′ and anti-sense: 5′-gaaaaactaataaaaaccagacaga- 3′) and unmethylated (sense: 5′-tttaggtatgtgaagtagggtagatgt-3′ and anti-sense: 5′-caaaaaactaataaaaaaccaacaaa-3′) with 180 pb as previously described [25] and 1 × Go Taq Hot Start Green Master Mix (Promega Corporation, Madison, WI, USA) for a final reaction volume of 25 μl. The PCR was conducted with an annealing temperature of 58 °C for 40 s and 40 cycles. Methylated and unmethylated DNA (Cells-to-CpG™ Methylated and Unmethylated DNA Control Kit, Life Technologies), which were modified, as previously mentioned, and amplified by PCR, as a control for the reactions and for the primers for the methylated and non-methylated conditions. Amplified PCR samples were loaded (7 μl) on 3% agarose gels with gel red and subjected to electrophoresis. The DNA bands were visualized with ultraviolet light.

### Statistical analysis

Frequencies were used for the categorical variables and descriptive statistics (mean, standard deviation) for the continuous variables. The Mann–Whitney U test and Chi Square tests and when necessary, and the Fisher Exact test were used, in the GraphPad program. Instat, version 3.0. For all tests, p < 0.05 was used as the significance level.

## Results

### Population characteristics

The epidemiological, clinical, anthropometric and metabolic characteristics of the studied groups are shown in Table 1. The DN group presented significantly higher frequencies of male (p = 0.0356), hypertensive (p < 0.0001) and dyslipidemic (p < 0.0001). Glycated hemoglobin was significantly higher in the DR group (p = 0.0306) than CONTROL group. Conversely, the creatinine parameters were significantly higher in DR (p = 0.0255) and DN (p = 0.0001), whereas microalbuminuria (p = 0.0001) and cholesterol (p = 0.0244) were higher in the DN group than CONTROL group. AGP was significantly higher in group CONTROL than DR group (p = 0.0172).

**Table 1:** Epidemiological, clinical, anthropometric and metabolic comparisons among the groups studied. Source: research data, 2017

	CONTROL (60)	DR (16)	*p*	DN (29)	*p*
Sex (M %)	26.6%	30.7%	0.4933	44%	0.0356*
Age (years)	57.5 ± 10	61 ± 9.1	0.3198	60 ± 6.8	0.2082
DM duration (years)	6 ± 2.1	7 ± 2.3	0.1092	7 ± 2.7	0.2000
Hypertention (%)	58.3%	69.2%	0.1089	92%	< 0.0001*
Dyslipidemia (%)	76.6%	69.2%	0.2391	96%	< 0.0001*
BMI (weight/height^2^)	28.7 ± 5.3	28.1 ± 4.1	0.5400	27.7 ± 4.5	0.4042
Abdominal circumference (centimeters)	104 ± 14.7	103 ± 11.3	0.7131	100 ± 10.7	0.2551
Glycemia (mg/dl)	152.5 ± 59.8	171 ± 62.5	0.6549	150 ± 53	0.8357
HbA_1_C (%)	7.8 ± 1.7	9 ± 2.6	0.0306**	8 ± 1.6	0.3880
Total cholesterol (mg/dl)	183.5 ± 42.9	189 ± 33.6	0.7293	193 ± 61.4	0.0244**
HDL cholesterol (mg/dl)	44 ± 10.6	44 ± 9.1	0.6037	41 ± 11.3	0.3853
LDL cholesterol (mg/dl)	105.5 ± 41.7	101.4 ± 49.6	0.9827	118 ± 61.3	0.1770
Triglycerides (mg/dl)	165.5 ± 93.2	177 ± 91.4	0.4578	177 ± 133.5	0.1252
Creatinine (mg/dl)	0.6 ± 0.3	0.8 ± 0.2	0.0255**	0.9 ± 0.3	0.0004**
Urinary albumin (mg/24 h)	5.2 ± 8.2	10.2 ± 8.1	0.2339	46 ± 158.1	0.0001**
CPR (mg/l)	2.4 ± 3.3	4.1 ± 3.3	0.1454	2 ± 3.3	0.8546
AGP (mg/dl)	80.5 ± 20.3	81 ± 29.8	0.6653	68 ± 31.4	0.0172**
TAC (%)	31.5 ± 30.2	44 ± 28.3	0.5072	13 ± 23.9	0.2116
MDA (µM)	3.7 ± 1	2.7 ± 1.5	0.1619	3.9 ± 1.2	0.5307

All comparisons were performed in relation to the CONTROL

* Significant difference—*X* ^2^

** Significant difference—Mann–Whitney U test

### DNA methylation and diabetic complications

**Fig. 1:**
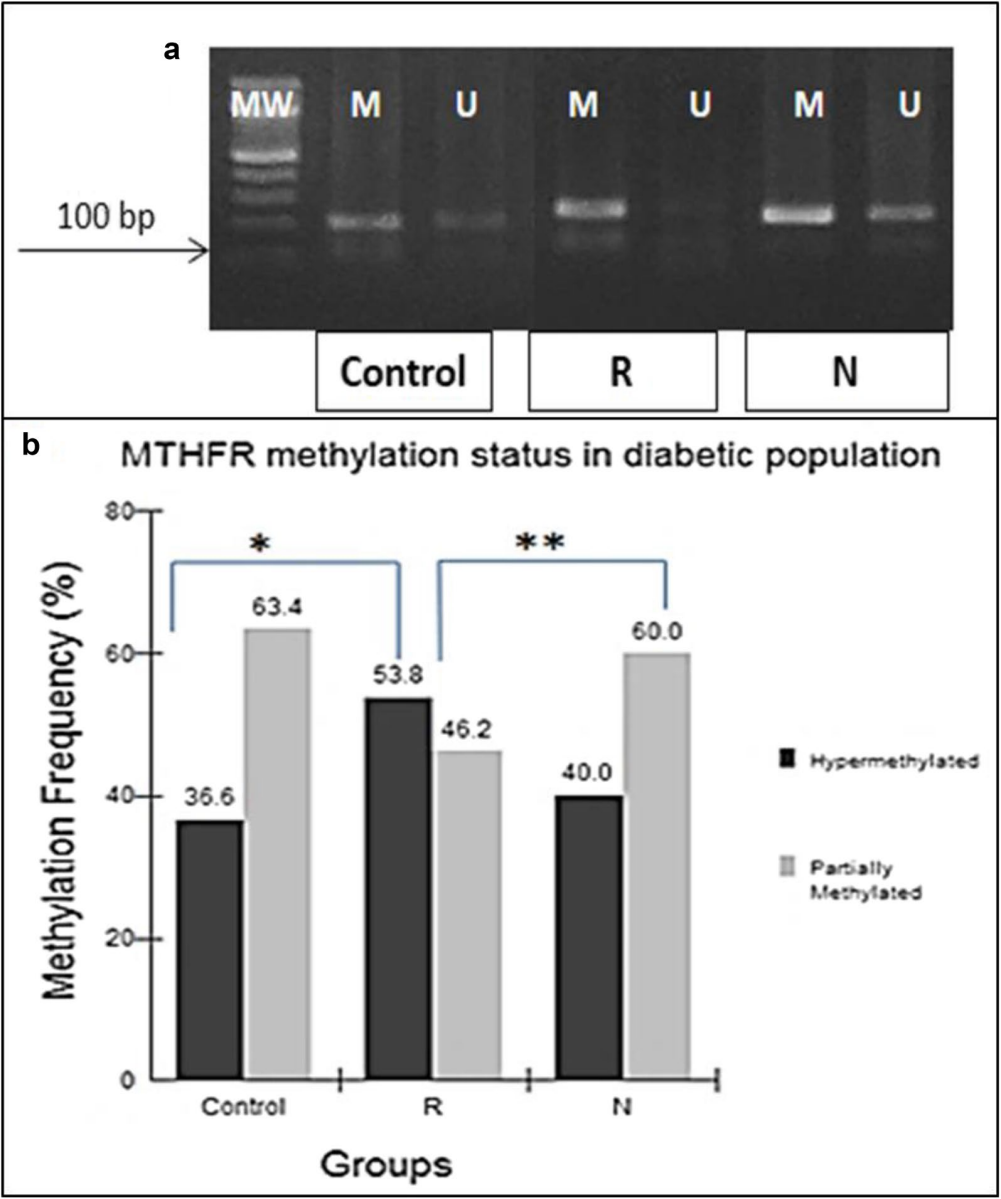
Methylation profile of DNA in the promoter of the MTHFR gene in patients with diabetes with and without complications. **a** Representative samples of the groups studied after MSP (180 bp). *MW* molecular weight, *M* methylated, *U* unmethylated. **b** Methylation frequency of Control (n = 60), DR (n = 16) and DN (n = 29) (*p < 0.01; **p < 0.03; Χ^2^). Partial methylation was defined as when the sample was positive for both methylated and unmethylated conditions (MSP)

The analysis of methylation in the promoter of the *MTHFR* for the studied samples showed that, 40.1% (43) had a hypermethylated profile, 59.8% (64) were partially methylated. The hypomethylated profile was not found (Fig. 1a). However, when stratifying the groups, it was observed that the DR group had a higher frequency of methylation (53.8%) when compared to the individuals in CONTROL (36.6%) and DN (40%) (*p = 0.0145; **p = 0.03; Χ2).

The comparison between the metabolic parameters and the methylation profile within the groups revealed significant differences for total cholesterol, LDL in the group with hypermethylated DR, TAC in the control and hypermethylated DN and AGP in the hypermethylated DR group (Table 2).

**Table 2:** Comparison of metabolic parameters according to methylation profile in the MTHFR gene. Source: research data, 2017

	CONTROL (60)			DR (16)			DN (29)		
	Hypermethylated	Partially methylated	*p*	Hypermethylated	Partially methylated	*p*	Hypermethylated	Partially methylated	*p*
Glycemia (mg/dl)	147.5 ± 56.3	158.5 ± 62.5	0.9267	183 ± 46.8	126.5 ± 74.3	0.1172	155 ± 55.5	150 ± 50.8	0.5686
HbA_1_C (%)	7.7 ± 1.3	7.9 ± 1.9	0.4615	8.5 ± 3	9.9 ± 2.4	0.4796	8.4 ± 1.8	7.9 ± 1.4	0.7924
Cholesterol (mg/dl)	169 ± 42.1	186 ± 43.9	0.6900	207.5 ± 30.1	165.5 ± 20.1	0.0103*	185 ± 65.3	199 ± 58.7	0.2875
HDL (mg/dl)	46.5 ± 9.9	43 ± 11	0.3654	44.5 ± 15.6	40.5 ± 9.5	0.2869	44 ± 8.5	38.5 ± 13	0.3804
LDL (mg/dl)	101.8 ± 39.4	107.9 ± 43.4	0.7185	111.5 ± 55.7	71.8 ± 28.7	0.0415*	103.6 ± 54.5	126 ± 59.7	0.1318
Triglyceride (mg/dl)	292 ± 69.5	159 ± 105.4	0.8121	184 ± 84.9	191 ± 134.7	0.4796	172 ± 169.2	176.5 ± 95.8	0.6816
Creatinine (mg/dl)	0.7 ± 0.2	0.6 ± 0.3	0.8539	0.8 ± 0.2	0.9 ± 0.2	0.2003	1.1 ± 0.3	0.9 ± 0.3	0.7756
Urinary albumin (mg/dl)	5.1 ± 7.7	5.6 ± 8.6	0.9449	11 ± 8.2	5.5 ± 9	0.3176	49 ± 166.2	45 ± 191.8	0.7588
CPR (mg/dL)	2.4 ± 3.1	2.4 ± 3.4	0.9939	4.3 ± 4.5	4.1 ± 3.1	0.3605	3 ± 2.5	1.8 ± 3.5	0.2875
AGP (mg/dl)	85.5 ± 20.3	76.5 ± 20	0.1604	85 ± 34.9	80.1 ± 36.4	0.3372	81 ± 15.4	63.5 ± 36.9	0.0080*
TAC (%)	57 ± 28.6	12.5 ± 28.1	0.0073*	64.5 ± 26.3	33.5 ± 24.5	0.1349	36 ± 26.3	12 ± 18.9	0.0169*
MDA (µM)	3.8 ± 0.6	3.6 ± 1.1	0.1106	2.6 ± 1.1	2.7 ± 1.9	0.4582	3.4 ± 1.3	3.7 ± 1.4	0.3456

* Significant difference—t test

** Significant difference—Mann–Whitney test

## Discussion

This work presents time as a factor in the control of diabetes time in the experimental groups. We consider this finding to be of great relevance, considering that both of the complications studied, DR and DN, tend to affect diabetic patients in a time dependent manner. Renal impairment may begin in the first decade of DM progression; DR may affect 60% of patients in the second decade of disease, even with glycemic control [26].

### Clinical and biochemical characteristics of the studied population

The DN group had a higher frequency of males with systemic arterial hypertension and dyslipidemia. Many diabetic patients have associated comorbidities, such as hypertension, and they may also present dyslipidemia, which is a risk factor for other chronic diseases. There is evidence that changes in blood pressure lead to urinary albumin excretion and, consequently, severe renal damage [27]. Evidence shows that dyslipidemia has a strong relationship with the microvascular complications of diabetes, especially nephropathy [28].

The comparison of biochemical parameters showed found significantly higher levels of HbA_1_C in the DR group. This finding is in agreement with previous studies that relate blood glucose control measured by the HbA_1_C levels and the risk of being affected by this complication [29]. It is not surprising that the relationship between DN, creatinine and microalbuminuria levels, was also observed patients with complications and without complications were compared, possibly because of the significance identified in the DN group. However, in this study, we did not observe a difference between microalbuminuria and DR, contrary to previous studies. We did observe a difference in creatinine levels [30].

Microalbuminuria was higher in the retinopathy and/or nephropathy and nephropathy groups, while creatinine was higher in the retinopathy and/or nephropathy, retinopathy and nephropathy groups. Although they are biomarkers of nephropathy, some studies also associate them with retinopathy [31]. It was found that glomerular filtration rates, even at low levels, in subjects with renal impairment were associated with the presence and severity of diabetic retinopathy compared to those with normal renal function [32]. Another study found that the progression of retinopathy is influenced by total cholesterol and creatinine.

CRP is an important marker of systemic inflammation, which has been physiologically related to atherosclerosis [33], pre-diabetes, is observed at high concentrations in neuropathic individuals [34] and correlates with increased macular thickness in patients with diabetic retinopathy [35]. In this study, we did not find a relationship between CRP, DN and DR, although in the second case, the values were close to the statistical limits of significance. The relationship with DR has been controversial. A study of diabetic Chinese population showed an inverse relationship between CRP values and DR [36], while a meta-analysis showed a relationship between the variables, suggesting that CRP can be used as a severity biomarker of retinal complications [37].

### DNA methylation and diabetic complications

The present study showed that the hypermethylated profile in the promoter of the *MTHFR* gene is associated with diabetic complications, particularly with retinopathy (Fig. 1). The group with DN showed a higher frequency of individuals with a partially methylated profile, similar to individuals with no complications. Partially methylated DNA indicates that methylation is not occurring in all alleles and/or leukocytes. A quantitative analysis could elucidate the percentage of methylation in these samples and may reveal differences between the group with no complications and the ND group.

A study of the Egyptian population identified *MTHFR* hypermethylation in the peripheral blood of patients with end-stage renal disease [38]. It is important to emphasize that the hypermethylation studied in the cited work was only samples that presented the partially methylated profile, since no fully methylated samples were found, unlike in the present study. On the other hand, the methylation that was found in our samples was not found in another study. The patient group in that consisted of individuals with renal disease, only 23% were diabetic; the control group consisted of healthy individuals, which changes the parameters of comparison compared to this study, because all patients in this study were diabetic.

Contrary to the methylation profile found in both the present work and in the Egyptian population, a recent study on a Chinese population showed a trend to hypomethylation of the *MTHFR* promoter in diabetic individuals with DN compared to healthy individuals [19]. In this same study, it was shown that diabetic patients without DN presented approximately 66.6% of methylated profile, although it is not clear whether it was total or partial methylation. Additionally, in this study, 36.6% of total methylation was found in diabetic subjects without complications. It is important to mention that the technique used and the CpG sites in all of these studies were the same, suggesting that these differences may be, primarily, the result of the demographic and clinical characteristics of the study population, which differed significantly between the studies. In addition, environmental factors that may be related to ethnicity-related habits, such as diet, may also influence the methylation profile of DNA.

Hypermethylation has also been identified in patients with ischemic stroke [39], preeclampsia [40], progenitors of people with Down syndrome [41], but it was not detected in patients with Non-Hodgkin’s Lymphoma. In male infertile patients with non-obstructive azoospermia, hypermethylation was identified in testicular samples, but not in peripheral blood [25].

As previously mentioned the CpG sites studied in the present work are located in a CpG island and are close to the binding sites for various transcription factors [20, 22]. Thus, hypermethylation of the gene could lead to decreased gene expression and consequently deregulate homocysteine reactions, leading to a decrease in the methyl radical and consequent epigenetic dysregulation due to the possible DNA hypomethylation. It has already been shown in cultured smooth muscle cells that demethylation was associated with increased levels of *MTHFR* transcripts [42].

### Correlation of the methylation profile at the MTHFR gene promoter with biochemical indicators of patients with diabetic complications

The hypermethylated profile is associated with a higher level of total cholesterol (p = 0.0103) and LDL cholesterol (p = 0.0415) in individuals with DR. The same association was identified in patients with end-stage renal disease. One possible explanation for this effect is that the increase in homocysteine resulting from low *MTHFR* expression (caused by hypermethylation in the gene promoter) would positively modulate cholesterol synthesis [43] since carbon metabolism has been described as the main donor of methyl groups [39].

In the present work, relationships between both total cholesterol and LDL with hypermethylated profile of *MTHFR* were obtained in patients with well-defined clinical profiles. It was not identified in patients with ND, and in diabetics without complications. These results suggest that the relationship is not simply be a consequence of Hcy metabolism but that other mechanisms that affect the synthesis or processing of cholesterol may be implicated and are present in patients with DR and those with end-stage renal disease.

In a review of lipid markers of DR that high levels of cholesterol and LDL were found to be associated with the development and severity of DR, especially in patients with formation of hard exudates in the retina [44]. This is probably due to the hyperglycemia that leads to oxidative stress and activates metabolic pathways of cell injury, especially AGE pathway, which generally increases with diabetes and is generated by non-enzymatic reactions of reducing sugars and lipoproteins. The effect of oxidative stress results in lipid peroxidation, production of malonaldehyde, damage to cell membranes and the accumulation of lipids [44–47].

The hypermethylated profile of the diabetic patients is also associated with serum AGP values and TAC. However, for AGP, the relationship was only observed in patients with DN and with complications with significantly higher levels in patients who have the methylated gene profile. AGP, also called orosomucoid is an acute phase protein produced in the liver under cytokine stimulation [48]. Levels of AGP have been related to parameters indicative of renal damage and the excretion of urinary albumin in diabetic patients [49] suggesting that it may be a predictor of the risk of this complication [50]. The relationship between *MTHFR* hypermethylation and increased levels of AGP may refine the screening of patients with potential renal complications.

For TAC, surprisingly, the relationship between hypermethylation and increased antioxidant capacity was evidenced in all groups except diabetic retinopathy. The same effect was not found in the MDA, a measure of lipid peroxidation. The relationship between the methylated profile of *MTHFR* and the increase in the levels of AGP and TAC remains to be clarified. It is possible that *MTHFR* hypermethylation leads to a decrease in gene expression, which would lead to global and/or site-specific hypomethylation, culminating in increased expression of several genes, including genes related to AGP synthesis and oxidative stress (TAC).

On the other hand, HbA1C, serum creatinine and urinary albumin, although altered in individuals with complications (Table 1), do not seem to be related to the methylation of the *MTHFR* gene, since after the correlation analysis between the methylation profile × biochemical indicators, they remained at similar levels in all groups (Table 2). This is easily explained, since diabetes is a multifactorial disease and, therefore, there is a combination of factors involved, in addition to the hypermethylation of *MTHFR.*

In summary, the present study analyzed the relationship between the methylation profile of the *MTHFR* gene with chronic complications of diabetes and the relation of this epigenetic mark with biochemical, inflammatory and oxidative stress parameters. It is the first analysis described in the literature with this approach that studies the gene in question for epigenetic aspects. It was performed in patients with time of finally managed diabetes in the phase in which the chronic complications usually start. We observed that the hypermethylated profile was the most frequent in individuals with DR and that this profile was associated with higher levels of total cholesterol and LDL. For patients with DN, although the hypermethylated profile did not predominate, it was associated with higher levels of AGP and TAC.

The primary weakness of the study is the reduced sample number, which is a consequence of the difficulty of recruiting individuals who fit within the inclusion criteria, especially regarding how long they have had diabetes. In addition, a quantitative analysis of the methylation profile could elucidate which proportion of the DNA from the partially methylated samples is methylated.

## Conclusions

The results of this study indicate that hypermethylation in the promoter of the *MTHFR* gene is associated with DR and biochemical, inflammatory, stress and oxidative parameters in the context of chronic complications of T2DM.

## Abbreviations

AGP: alpha-1 acid glycoprotein
CpG: cytosine/phosphodiester binding/guanine
CRP: C-reactive protein
DM: diabetes mellitus
T2DM: type 2 diabetes mellitus
DN: diabetic nephropathy
DR: diabetic retinopathy
HbA1c: glycosylated hemoglobin
Hcy: homocysteine
HDL: high density lipoprotein
HHcy: hyperhomocysteinemia
LDL: low density lipoprotein
MBP: methyl binding proteins
MDA: malondialdehyde
MTHFR: 5,10-methylene tetrahydrofolate reductase (enzyme)
*MTHFR*: gene that codes the MTHFR
pb: base pairs (of nucleotides)
PCR-MSP: polymerase chain reaction specific for methylation
PRIMER: primer oligonucleotide
SAMe: S-adenosylmethionine
TAC: total antioxidant capacity
